# Preserving the Flame: The Past, Present, and Future of EJOP

**DOI:** 10.5964/ejop.11945

**Published:** 2023-05-31

**Authors:** Johannes Karl

**Affiliations:** 1 Dublin City University, Dublin, Ireland

Europe’s Journal of Psychology has a long history of pushing boundaries in psychological publishing, being an early journal adopting a diamond open access model, allowing for the sharing of information to a wide range of audiences by a wide range of authors. As I am taking over the editorship of this journal, I want to continue this legacy and continue to push boundaries with this journal. With this will come a number of changes that aim to stimulate new ways of how we do research. These changes can be summarized under three key points: Exploration, Replication, and Reflection. Since the inception of EJOP it has published more than 800 articles from 1458 unique authors across 70 countries. This represents a substantial level of diversity, which is made even more compelling by the fact that nearly one fifth of all articles in EJOP have been authored by cross-national author teams. In the future we want to carry forward this diversity, specifically encouraging submissions from areas historically underrepresented in psychological journals (Henrich, 2020).

Raising the unexpected, curious, and thought provoking to the eye of the scientific community is in my opinion one of the cornerstones of the advancement of science. By being presented with empirical observations that make us question our held beliefs we can grow simultaneously as individual researchers and as scientific community. While a large focus since the replications crisis in psychology has been ensuring the robustness of the cumulative psychological corpus (Lilienfeld, 2017; Shrout & Rodgers, 2018), many researchers have highlighted that attempts at replication need to go hand in hand with an open curious exploration of novel phenomena (Fife & Rodgers, 2022).

In line with this come the first two concrete changes for EJOP that will be relevant for all authors going forward. First, we now require all articles to fulfill Level 3 of our Open Science practices as outlined on the EJOP website. In practices this means that all studies submitted for review in EJOP require their data, code, and materials to be made available in a form which allows other researchers the ability for unrestricted access and reuse with proper attribution. This means we will no longer publish quantitative studies which do not allow for computational replication of a study without input from the original authors. To support authors and reviewers in ensuring the highest quality of their data, data-dictionary, and code as well as the plain language statements, we will create additional junior editor positions in EJOP who will oversee the application of these processes, for which we encourage applications to the editor. We are cognizant of the heterogeneity of research approaches and fields, especially in qualitative research (Prosser et al., 2022). We therefore encourage authors who aim to submit an article which contains data that for legal, ethical, or other reasons cannot be made public at the moment of submission to contact the editor in advance of submission to negotiate alternative solutions (such as time-delayed release or alternative approaches to anonymization).

Second, notwithstanding the previous point about the value of curious exploration as basis of scientific progress, to build psychology as a discipline we require a modicum of understanding how robust our findings are and what are the boundary conditions of our observed effects. While much has been written in the main-stream psychological literature about the failures to replicate highly visible studies these approaches only cover a small sub-section of our total field. Even more, it is often unclear why an effect was not replicated; was there genuinely no effect, was the observed effect in the original study time-bound or culture bound? Without systematic investigation of the limits of our own effects and systematic study what determines these limits it is highly likely that psychology will continue to deliver unreliable findings or will be forced to substantively reduce its scope of investigation.

With this in mind come the second set of concrete changes to EJOP. We now invite registered replications of published psychological research. Authors can either pre-register at any reputable repository of choice or authors can submit a registered report to EJOP which will be reviewed and can be accepted in principle prior to the commencement of the study. To support coherent coverage of the psychological literature (for a review see Makel et al., 2012) we will place primacy on manuscripts reporting replication efforts of studies which have not received replication efforts prior. Further, we are explicitly inviting papers that systematically examine the replicability of established psychological constructs or effects across both time and cultural boundaries.

Third, while we believe that these aforementioned changes are necessary to allow psychology to grow as discipline, we are aware of the existing global and institutional inequity in access to high quality information on theory and application of existing and novel methods. We want to support efforts in narrowing this gap allowing for equal participation of all researchers in the advancement of psychology. Therefore, we invite tutorials, practical guides, and detailed reflections aimed at methodology in psychological research, both about research procedures (such as experimental procedures and psychometric measures) as well as about statistical techniques and advances. We will place an especially strong focus on publications which support readers in the practical implementation, beyond theoretical considerations, with code, protocols, and other concrete implementations. We believe that EJOP as a Diamond Open-Access Journal is uniquely placed to allow both authors and readers to disseminate and access this crucial information without the barriers often faced by many researchers aiming to access information in the literature.

Finally, in line with our mission to make psychological science accessible to all researchers, either as readers or as authors, we also want to ensure that research in EJOP is accessible to the public. While our Diamond Open Access model allows interested general public readers access without traditional barriers, this does not mitigate the barriers often experienced through lacking accessibility of the scientific content. This lack of accessibility can lead to the spread of misinformation based on published articles which are mischaracterized or reduce the informational impact science can have on the general public. To address this, we will we now require all published studies to prepare a plain language statement in addition to an abstract which summarizes the research in a form accessible to the general public. We hope that by providing research in both a way that is without monetary barriers and accessible to a broader public we can support a more evidence based public discourse. To support authors in the creation of these plain language statements we will create junior editor positions who will support and oversee this process. For these positions we explicitly encourage applications especially from researchers and practitioners with a background in communicating science effectively.

Overall, our hope is that these changes will help EJOP to continue to grow, fulfilling its legacy of a journal open to all, while also addressing the new challenges psychology and the wider society haven been facing since the near 20 year of its inception.

## References

[r1] Fife, D. A., & Rodgers, J. L. (2022). Understanding the exploratory/confirmatory data analysis continuum: Moving beyond the “replication crisis.” American Psychologist*,* 77, 453–466. 10.1037/amp0000886 34780242

[r2] Henrich, J. (2020). *The WEIRDest people in the world: How the west became psychologically peculiar and particularly prosperous*. Farrar, Straus and Giroux.

[r3] Lilienfeld, S. O. (2017). Psychology’s replication crisis and the grant culture: Righting the ship. Perspectives on Psychological Science*,* 12(4), 660–664. 10.1177/1745691616687745 28727961

[r4] Makel, M. C., Plucker, J. A., & Hegarty, B. (2012). Replications in psychology research: How often do they really occur? Perspectives on Psychological Science*,* 7(6), 537–542. 10.1177/1745691612460688 26168110

[r5] Prosser, A. M. B., Hamshaw, R. J. T., Meyer, J., Bagnall, R., Blackwood, L., Huysamen, M., Jordan, A., Vasileiou, K., & Walter, Z. (2022). When open data closes the door: A critical examination of the past, present and the potential future for open data guidelines in journals. British Journal of Social Psychology. 10.1111/bjso.12576 36076340 PMC10946880

[r6] Shrout, P. E., & Rodgers, J. L. (2018). Psychology, science, and knowledge construction: Broadening perspectives from the replication crisis. Annual Review of Psychology*,* 69(1), 487–510. 10.1146/annurev-psych-122216-011845 29300688

